# New anti-cancer explorations based on metal ions

**DOI:** 10.1186/s12951-022-01661-w

**Published:** 2022-10-23

**Authors:** Han Hu, Qi Xu, Zhimin Mo, Xiaoxi Hu, Qianyuan He, Zhanjie Zhang, Zushun Xu

**Affiliations:** 1 Ministry of Education Key Laboratory for the Green Preparation and Application of Functional Materials, Hubei Key Laboratory of Polymer Materials, School of Materials Science and Engineering, Hubei University, Wuhan, 430062 Hubei China; 2 Cancer Center, Union Hospital, Tongji Medical College, Huazhong University of Science and Technology, Wuhan, 430022 China; 3 College of Petroleum and Chemical Engineering, Beibu Gulf University, Qinzhou, 535011 China

**Keywords:** Metal ions, Cell death, Cancer therapy, Diagnosis, Bioimaging, Nano-theranostic

## Abstract

Due to the urgent demand for more anti-cancer methods, the new applications of metal ions in cancer have attracted increasing attention. Especially the three kinds of the new mode of cell death, including ferroptosis, calcicoptosis, and cuproptosis, are of great concern. Meanwhile, many metal ions have been found to induce cell death through different approaches, such as interfering with osmotic pressure, triggering biocatalysis, activating immune pathways, and generating the prooxidant effect. Therefore, varieties of new strategies based on the above approaches have been studied and applied for anti-cancer applications. Moreover, many contrast agents based on metal ions have gradually become the core components of the bioimaging technologies, such as MRI, CT, and fluorescence imaging, which exhibit guiding significance for cancer diagnosis. Besides, the new nano-theranostic platforms based on metal ions have experimentally shown efficient response to endogenous and exogenous stimuli, which realizes simultaneous cancer therapy and diagnosis through a more controlled nano-system. However, most metal-based agents have still been in the early stages, and controlled clinical trials are necessary to confirm or not the current expectations. This article will focus on these new explorations based on metal ions, hoping to provide some theoretical support for more anti-cancer ideas.

## Introduction

Cancer is one of the problems of severe threats to human life and health. Traditional surgical therapy has excellent trauma and narrow application scope. Radiation therapy and chemotherapy cannot effectively distinguish between normal and cancerous cells, inevitably bringing severe side effects on normal tissues and organs [1]. Moreover, the more dangerous is the possibility of cancer metastasis and recurrence [2]. Therefore, there is an urgent need to find new anti-cancer methods to overcome these obstacles as much as possible.

Metal ions significantly impact the biosystem and play an essential role in diverse physiological activities such as maintaining cell homeostasis, regulating metabolic pathways, substance synthesis, signal transmission, and energy conversion. [3]. In studying the biological behavior of metal ions, researchers have found that abnormal distribution of metal ions affect the various physiological function of cells, causing adverse effects and even death [4–6]. These findings suggest that metal ions have specific mechanisms for inducing cell death, which makes related strategies have been soon studied in cancer treatment. In the past few decades, although many anti-tumor drugs based on metal ions have been designed and synthesized, except for some Pt-containing anti-tumor agents that show effective therapeutic effects, most of the agents are still in the early stages and fail to achieve broader clinical practice [4, 7]. Therefore, researchers are eager to explore more new applications of metal ions in cancer therapy.

On the other hand, the cancer diagnosis can intuitively show the feature information of the tumor [8], which can be conducive to guiding treatment. The bioimaging technology applied in cancer diagnosis usually requires the assistance of contrast agents [9, 10]. Metal ions have previously been used in imaging technology, such as the contrast agents based on Gd^3+^, which have dominated the MRI field for several decades [11]. However, the problems of traditional contrast agents limit their prospects for biological applications [12], such as insufficient time in vivo circulation of iodine-based and barium-based agents [13], the short half-life period of radioactive ^18^F [14], and the high toxicity of gadolinium ions [15]. Therefore, researchers are actively exploring new alternatives, and the new nanoprobes designed by metal ions with more specific physical and chemical characteristics have developed promptly.

**Fig. 1:**
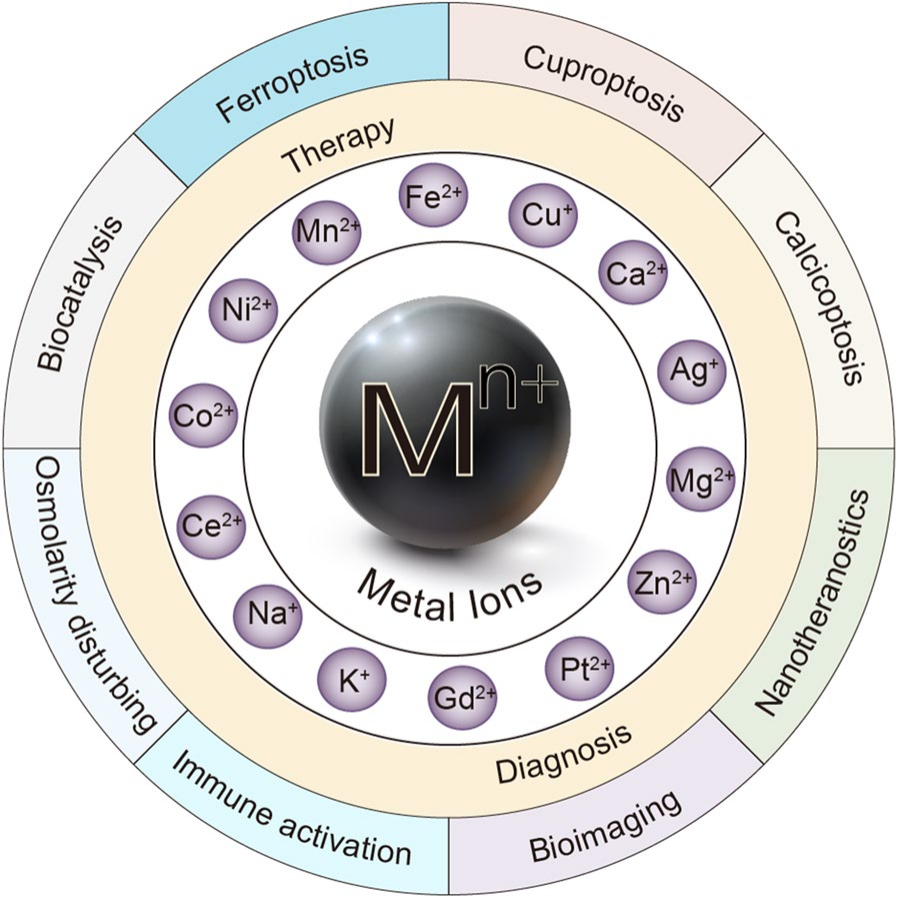
New applications of metal ions in cancer therapy and diagnosis

Nanomaterials have recently attracted significant attention in the biomedical field due to their unique thermal, optical, electrical, and magnetic properties [16]. They can enter tumor cells in particular ways, which has enormous implications for drug delivery [17, 18]. While first-generation nanoparticles offered considerable promise in cancer therapy and diagnosis, toxicity and non-specific distribution hindered their true potential [19]. Therefore, the new nano-theranostic platform based on metal ions may serve to plan alternative therapeutic strategies, possibly with lower biotoxicity, higher responsiveness, and controllability. This review will summarize the new anti-cancer applications of metal ions from two aspects of therapy and diagnosis (Fig. 1).

## New approaches to metal ions-induced cell death and their anti-cancer applications

With the study of the relation between metal ions and cytology deepening in recent years, many metal ions have been found to possess cell-death-inducing effects. Typically, ferroptosis is still the research hotspot, with more relevant anti-tumor strategies increasing over the years. Newly proposed calcicoptosis and cuproptosis have also rapidly become the focus in the cancer field. Amazingly, more metal ions have been shown to induce cell death in different approaches, contributing to more breakthroughs in internal cancer therapy. This chapter will summarize the mechanisms and anti-tumor applications of these new approaches.

### Ferroptosis

**Fig. 2:**
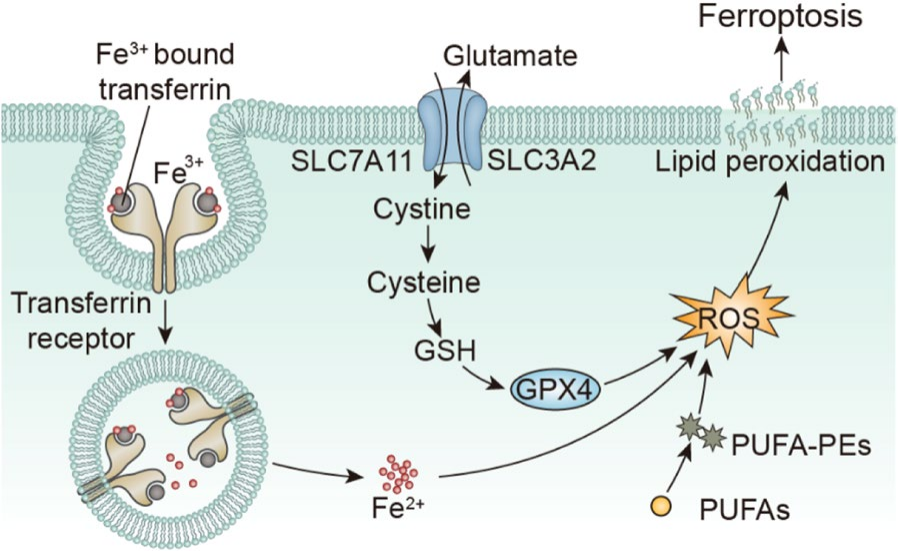
Schematic illustration of ferroptosis mechanism based on lipid peroxidation [33]

Iron ions are significant transition metal ions in organisms, and they show high internal content and regulate many aspects of cell metabolism [20, 21]. Ferroptosis is proposed by Dr. Brent R. Stockwell's team, which is different from apoptosis and necrosis, and characterized by the accumulation of iron-dependent lipid peroxidation (Fig. 2) [22, 23]. In recent years, ferroptosis has aroused great interest from cancer researchers as a unique mechanism of cell death. Bracingly, substantial progress has been made in the research of ferroptosis in tumor biology and cancer therapy. Varieties of cancer-related signaling pathways have been proved to control the ferroptosis of cancer cells, and the peculiar metabolic mechanism of the tumor makes some of them inherently prone to ferroptosis, thus exposing the weakness that they can be utilized as therapeutic targets in some cancer types [24].

The occurrence and execution of ferroptosis mainly depend on various unique metabolic mechanisms [25–27]. Transferrin (TF) has a high affinity with Fe^3+^ [28]. After binding with transferrin (TF), Fe^3+^ can enter cells through transferrin receptor 1 (TFR1) [29]. Meanwhile, the Six-Transmembrane Epithelial Antigen of Prostate 3 (STEAP3) can reduce Fe^3+^ to unstable and catalytic Fe^2+^ [30]. When Fe^2+^ overload exceeds the ferritin buffer capacity, nuclear receptor coactivator 4 (NCOA4) will degrade it, releasing a large amount of Fe^2+^ into the cytoplasm [31], triggering the Fenton reaction to generate a large number of hydroxyl radicals (·OH) with high oxidation activity. That will cause irreversible oxidative damage to cells. When ROS production exceeds the scavenging capacity of the cell’s antioxidant system, accumulation of lipid peroxides will be generated [32], further promoting ferroptosis in cells.

Excessive oxidation of phospholipids in polyunsaturated fatty acids (PUFAs) also contributes to cell ferroptosis [34]. PUFAs are an essential component of the phospholipid bilayer of the cell membrane and play an indispensable role in maintaining cell membrane fluidity [35]. Studies have shown that the abundance of PUFAs determines the degree of lipid peroxidation [36]. When PUFAs are overexpressed, Fe^2+^ will be oxidized into many lipid peroxides through the Fenton reaction to induce ferroptosis in cells [37]. Lipidomics shows that the Phosphatidylethanolamine (PEs) esterified by arachidonic acid (AA) and adrenal acid (AdA) are the primary substrates for peroxidation in the process of ferroptosis [38]. Long-chain acyl-CoA synthetase 4 (ACSL4) activates AA and AdA to arachidonic acid-CoA and adrenal acid-CoA. These derivatives are then esterified into PEs by lysophosphatidylcholine acyltransferase 3 (LPCAT3) and enter membrane phospholipids. PEs will be oxidized to lipid peroxides by lipoxygenase (LOXs), thus triggering ferroptosis in cells [39].

System Xc-, an intracellular cysteine-glutamate exchange transporter, is also one of the critical targets for inducing ferroptosis [40]. System Xc- is a dimer that connects the light chain subunit (xCT, SLC7A11) and heavy chain subunit (CD98hc, SLC3A2) through disulfide bonds. SLC7A11 is the active part of the transporter, while SLC3A2 is responsible for regulating the transport function on the cell membrane [41, 42]. System Xc- is located in the cell membrane and is mainly in charge of the uptake of cystine and the excretion of glutamate in the cell [43]. Cystine is transformed into cysteine after transport into cells, and glutathione (GSH) which can protect cells from oxidative damage, is synthesized to provide antioxidant defense for cells and prevent excessive accumulation of intracellular lipid peroxides [44]. Glutathione peroxidase 4 (GPX4) is located downstream of System Xc-, which can catalyze GSH degradation of small molecule peroxides and some lipid peroxides [45]. However, the depressed expression of GSH will directly decrease the activity of GPX4 [46]. Therefore, inhibition of GSH and GPX4 expression also leads to the accumulation of lipid peroxides, inducing ferroptosis.

Based on various primary mechanisms above, nanomaterials that can trigger ferroptosis have been designed and applied in cancer treatment (Table.1) [47]. Iron ions can generate toxic hydroxyl radicals through the Fenton reaction, and the accumulation of hydroxyl radicals will induce ferroptosis [48]. Therefore, the concentration of iron ions in cells and the expression level of H_2_O_2_ are crucial to the above process. Many biological nanomaterials have been designed to induce ferroptosis in tumor cells by increasing the concentration of Fenton substrates such as iron ions and H_2_O_2_. Shen et al. [49] successfully transferred Fe_3_O_4_ and Gd_2_O_3_ loaded with cisplatin (CDDP) to the tumor cell by nanoparticles. Released iron ions could directly participate in the Fenton reaction, while CDDP would indirectly produce H_2_O_2_. Accelerate the Fenton reaction to produce reactive oxygen species and induce ferroptosis in tumor cells. Wan et al. [50] designed an iron-metal–organic framework (MOF) coated with a cancer cell membrane decorated with glucose oxidase (GOx) as a nano-drug. When the drug reached the tumor site, the high concentration of GSH would cause the structural collapse of MOF and release Fe^2+^ by reducing Fe^3+^. GOx could catalyze glucose to produce H_2_O_2_, which promoted the occurrence of the Fenton reaction and accelerated the production of ·OH. Liang et al. [51] synthesized polyethylene iron-Copper MOFs (FCSP MOFs) as ferroptosis inducers and grew gold nanoparticles on FCSP MOFs in situ. Au NPs could transform excess glucose intake into gluconic acid and H_2_O_2_ in tumor cells and trigger the coordinated Fenton reaction by iron and copper ions. In vitro and in vivo experiments showed that this MOF material could effectively induce ferroptosis and significantly improve the treatment efficiency of radiotherapy. The above typical examples show that increasing the concentration of Fenton-triggering substrates in tumor cells can accelerate the accumulation of hydroxyl radicals, significantly enhancing ferroptosis's efficacy.

**Table 1:** Strategies to design ferroptosis-inducing nanomaterial for cancer therapy

Strategy	Nanomaterial example	Ferroptosis components	References
Producing reactive oxygen by Fenton reaction	Fe_3_O_4_/Gd_2_O_3_@CDDP@LF/RGD2	Fe_3_O_4_, CDDP	[49]
	Gox@MOF(Fe)@CCM	Fe^2+^, Fe^3+^, GOx	[50]
	AuFCSP MOFs	Fe^2+^, Fe^3+^, Au NPs	[51]
Inhibiting the activity of GXP4	RSL3@COF-Fc	Fc, RSL3	[56]
	HMPB/ ML210@TA-BLM-Fe^3+^	Fe^2+^, Fe^3+^, ML210	[57]
Consuming intracellular GSH	CA-OH-Fe^3+^/Gd^3+^@P-SS-D	Fe^2+^, Fe^3+^, CA	[58]
	siMCT4-PAMAM-PEG-TK-Fc@DEM	Fc, DEM, siMCT4	[59]
	FPBC@SN	Fe^2+^, Fe^3+^, SRF, NLG919	[60]
Inhibiting the activity of System Xc-	Ce6-Erastin	Erastin	[66]
	SSZ-Fe^2+^@DSSD	Fe^2+^, SSZ	[67]
	MIL-101(Fe)@SRF	Fe^2+^, Fe^3+^, SRF	[68]
Delivering exogenous lipid peroxides	IO-LAHP	IO NPs, LAHP	[71]
	RSL3@mPEG-PLys-AA	RSL3, AA	[72]
	Lecithin@FAC	Fe^2+^, Fe^3+^, Lecithin	[73]

GPX4 is a selenoprotein that catalyzes glutathione to convert lipid peroxides into lipid alcohols [52], effectively removing the accumulation of lipid peroxides in cells and providing antioxidant defense against ferroptosis [53]. GSH is the most crucial cofactor of GPX4, and the expression level of GSH will directly affect the activity of GPX4. Nano-therapy that inhibits GPX4 activity has also been used to induce ferroptosis. The strategy of this therapy is mainly to directly deliver nano-drugs with GPX4-inhibiting function to the tumor cell or inhibit GPX4 activity by efficiently removing GSH or blocking its synthesis [54]. Various GPX4 inhibitors, such as RSL3, ML162, and ML210 [55], have been developed, providing more explicit directions for strategies to inhibit GPX4 activity. Zhou et al. [56] prepared RSL3@COF-Fc NPs. RSL3 is an effective ferroptosis inducer, which could specifically inhibit the expression of GPX4. At the same time, ferrocene would trigger the Fenton reaction to produce ·OH in cells. The synergistic action of these two methods led to the massive accumulation of lipid peroxides. Zhou et al. [57] designed and prepared HMPB/ML210@TA-BLM-Fe^3+^ nano-composites. After degradation in tumor cells, it would release ML210, effectively inhibiting the activity of GPX4 and activating the ferroptosis pathway. The Fenton reaction induced by iron ions dramatically upregulated intracellular reactive oxygen species, which led to the accumulation of lipid peroxidation. In vivo anti-tumor experiments also showed that the nanomaterials could be used as effective ferroptosis inducers.

Moreover, the strategies that indirectly inhibit GPX4 activity by down-regulating the expression of GSH are relatively more. Luo et al. [58] have fabricated a theranostic nano-platform (FCS/GCS). The nanoparticles would depolymerize because of the high GSH expression in the tumor microenvironment (TME), and the activated cinnamaldehyde (CA) could consume GSH and down-regulate GPX4. And then, the Fenton reaction produced abundant ·OH and accelerated the accumulation of lipid peroxides, thus enhancing ferroptosis. In Zhang’s experimental system [59], the diethyl maleate (DEM) could directly reflect the consumption of GSH, thereby destroying GPX4-mediated antioxidant defense, and siMCT4 would block MCT4-mediated lactic acid excretion to acidify the intracellular environment, which improved lipid peroxidation induced by ferrocene. Zuo et al. [60] designed and synthesized FPBC@SN NPs decomposed and released ferritin, sorafenib (SRF), and an IDO inhibitor (NLG919) in acidic cytoplasm. NLG919 stimulated anti-tumor immunity by inhibiting IDO and reducing tryptophan metabolism. SRF has been used as a chemotherapy drug in a few clinical trials and could also block glutathione synthesis and down-regulated GPX4. Meanwhile, iron ions obtained by ferritin degradation would participate in the Fenton reaction to generate lipid peroxides to promote ferroptosis in tumor cells.

System Xc- is an essential component of antioxidants in cells [61]. System Xc- activity is generally positively correlated with the expression level of the light chain encoded by SLC7A11 [62]. SLC7A11 is overexpressed in various cancers; it promotes glutathione synthesis, providing antioxidant defense for tumor cells by introducing cysteine [63]. Recent studies have shown that SLC7A11 could partially promote tumor growth by inhibiting ferroptosis [64], so inhibition of System Xc- activity to induce ferroptosis in tumor cells has gradually become a feasible strategy. Some System Xc- inhibitors such as Erastin, SSZ, and Sorafenib have been applied to cancer. [65]. Zhu et al. [66] have designed a novel nano-medicine by self-assembling between photosensitizer chlorin e6 (Ce6) and Erastin. Erastin reduced intracellular GSH concentration by inhibiting cysteine uptake, inducing ferroptosis in tumor cells. At the same time, ROS produced by Ce6-guided photodynamic therapy (PDT) could be further accumulated to achieve the effect of enhancing PDT. Xin et al. [67] successfully synthesized the SSZ-Fe^2+^@DSSD nano-drug. When a high level of glutathione destroyed the disulfide bond in the system, Sulfasalazine (SSZ) and Fe^2+^ could be released. SSZ could induce ferroptosis by inhibiting the activity of System Xc-, and Fe^2+^ would trigger the Fenton reaction. The experiment results showed that the nano-drugs could synergistically improve the therapeutic effect of ferroptosis in two ways. Liu et al. [68] constructed MIL-101(Fe) nanoparticles loaded with Sorafenib (SRF) and evaluated the therapeutic effect. After co-administration with iRGD peptide in vitro and in vivo, MIL-101(Fe)@SRF NPs significantly induced ferroptosis and decreased the expression of GSH and GPX4, and increased lipid peroxidation levels in HepG2 cells. However, comparative studies have also indicated that these System Xc- inhibitors have failed to trigger ferroptosis in a wide range of cancer cell lines, suggesting that some cancer cell lines seem resistant to inhibition of System Xc- [65]. Thus, the related biological mechanisms need to be further studied.

The abundance of polyunsaturated fatty acids greatly affected intracellular lipid peroxidation [69]. However, the substrate of lipid peroxidation is usually endogenous [70]. Therefore, it may be feasible to induce ferroptosis by delivering exogenous lipid peroxides through nanomaterials. Many unsaturated lipids such as arachidonic acid, linoleic acid, phosphatidylcholine, and linolenic acid have been used as the exogenous initiators of ferroptosis, which achieved gratifying curative effect. Zhou et al. [71] prepared iron oxide nanoparticles modified by linoleic acid peroxide (LAHP) and realized the corresponding release of Fe^2+^ under the acidic TME to trigger the Fenton reaction. The exogenous introduction of LAHP increased the degree of lipid peroxidation in tumor cells and promoted the accumulation of lipid ROS, which caused irreversible oxidative damage to tumor cells. Gao et al. [72] successfully synthesized an amphiphilic polymer nano-micelle RSL3@mPEG-PLys-AA. Exogenous arachidonic acid significantly increased the level of intracellular lipid peroxides, which could trigger ferroptosis. Under the action of free radicals, micelles would disintegrate and rapidly release RSL3, which could inhibit GPX4 activity and promote the accumulation of lipid peroxides. He et al. [73] have prepared a novel nano-reactor to generate LPO. The LPOgener comprised ammonium ferric citrate (FAC) and phosphatidylcholine rich in unsaturated lipids, and Fe^3+^ was encapsulated. Under the action of GSH, Fe^3+^ could be effectively reduced to Fe^2+^, and the oxidation–reduction of GSH and iron ions would trigger the continuous release of unsaturated lipids from LPOgener, thus inducing the ferroptosis. The study also provided ideas for designing new anti-tumor strategies.

To sum up, ferroptosis mainly relies on iron metabolism, lipid metabolism, and antioxidant metabolism, resulting in a significant accumulation of lipid peroxides in tumor cells and thus inducing cell death [74]. Therefore, various strategies to induce ferroptosis have been applied to cancer treatment, including triggering Fenton reaction by accumulating iron ions, inhibiting GPX4 activity or reducing the expression of GSH, inhibiting System Xc- activity, and exogenous delivery of lipid peroxide. All of the above strategies can promote the accumulation of lipid peroxides in tumor cells. Nowadays, ferroptosis has still been the research hotspot in cancer, and it is believed that ferroptosis therapy will become a more mature means against cancer.

### Calcicoptosis

The role of calcium ions (Ca^2+^) in biological systems is unique, usually acting as the messenger of intracellular signal transmission [75, 76]. Many functions of cells depend on the change of Ca^2+^ concentration in the cytoplasm, and once the concentration changes out of control, cell functions will be interfered with and even cause cell death [77, 78]. As is known to all, after a period of chemotherapy and radiotherapy, the CT images of tumor lesions in patients with significant therapeutic effects will show calcification spots [79, 80]. This phenomenon cannot help but make people consider whether there is a necessary relationship between the calcification development of tumor lesions and the therapeutic effect.

From the perspective of cell biology, radiotherapy and chemotherapy in the tumor lesion area can lead to oxidative stress response to induce calcium overload of tumor cells, thus forming calcification points in the lesion area and ultimately leading to tumor cell death [81]. Relevant studies have shown that under normal circumstances, cells have elaborate regulation mechanisms (endoplasmic reticulum (ER), calcium pump, Ca^2+^ channel) for Ca^2+^ concentration, making cellular calcium overload difficult to occur [82, 83]. However, under oxidative stress, the regulatory ability of cells to Ca^2+^ will gradually decline, resulting in the Ca^2+^ continuous accumulation and calcium overload [84], which suggests a synergistic effect between cellular oxidative stress and calcium overload.

**Fig. 3:**
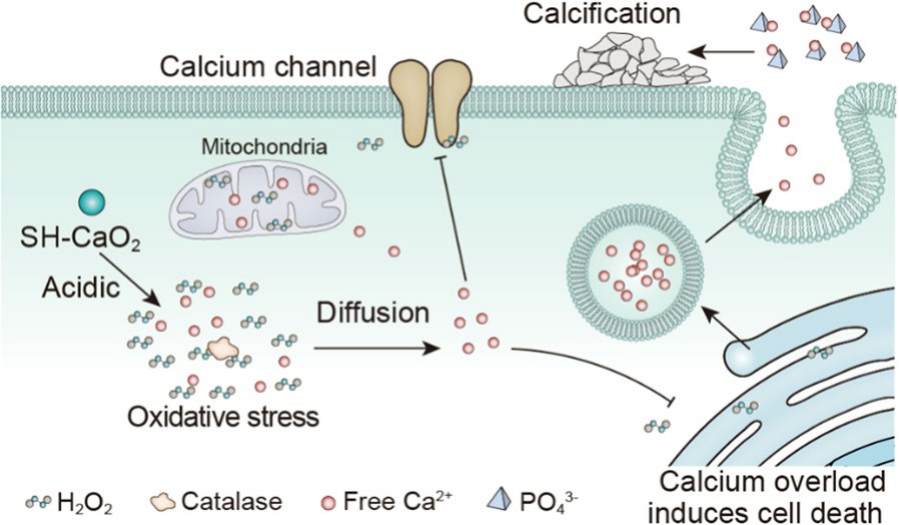
Schematic illustration that calcium overload induces tumor cell calcicoptosis [85]

Therefore, inspired by the calcification point phenomenon of clinical tumors, Bu's team combined this clinical medical phenomenon with material science to induce calcium overload in tumor cells through calcium-based nanomaterials for combined therapy [85]. The collaborative research team ingeniously designed and synthesized a class of CaO_2_ nanoparticles with a particle size of less than 5 nm and coated a layer of pH-sensitive sodium hyaluronate (SH) on their surface to obtain ultra-small SH-CaO_2_ nanoparticles. In the acidic TME, the protective layer of hyaluronic acid was decomposed, and the exposed CaO_2_ accelerates decomposition to produce H_2_O_2_ and free Ca^2+^ under acidic conditions. Accumulation of H_2_O_2_ induced oxidative stress in tumor cells, which led to Ca^2+^ channel dysfunction and impeded normal regulation of Ca^2+^ concentration within tumor cells, resulting in the long-term retention of Ca^2+^ in tumor cells and causing persistent cellular calcium overload. The metabolic and proliferative processes of tumor cells would be interfered with, thus inducing cell death. At the same time, local enrichment of Ca^2+^ can also promote the development of calcification in tumor lesions. From the perspective of materialogy, the main component of calcification in tumor lesions was hydroxyapatite. This type of tumor cell death was defined in this study as calcicoptosis (Fig. 3).

Calcicoptosis has been associated with many cancer treatments in recent years. Due to the unique regulation mechanism of Ca^2+^ in cells, multi-mode treatment strategies based on calcicoptosis have been paid more attention. Liu's team synthesized a CaO_2_ and copper peroxide nano-composite modified by hyaluronic acid through a simple one-step strategy [86]. After the effective accumulation of nanomaterials at the tumor site, the TME with low pH and overexpression of hyaluronidase can induce the simultaneous release of large amounts of Ca^2+^, Cu^2+^, and H_2_O_2_. Thus, oxidative stress of tumor cells was enhanced, calcium transport imbalance was further caused, and calcium apoptosis was induced in tumor cells. In addition, the synergistic effects of Fenton-like responses triggered by Cu^2+^ and mitochondrial dysfunction induced by calcium overload also enhance the combination therapy of calcicoptosis and chemodynamic therapy (CDT). Zheng's team prepared an acid-sensitive, polyethylene glycol-modified calcium carbonate (CaCO_3_) nanoparticle containing curcumin (CUR; a Ca^2+^ enhancer) by a one-pot method [87]. The nanoparticles can be used as a nano regulator to induce mitochondrial calcium overload and as an immunogenic cell death (ICD) inducer to achieve calcicoptosis while triggering a robust anti-tumor immune response. Tan et al. reported a core–shell structure nano-ultrasonic sensitization agent (TiO_2_@CaP) [88], which could not only reactivate the production of reactive oxygen species (ROS) but also dissolve its CaP shell and release Ca^2+^ in the ultrasonic activated acidic microenvironment, intracellular oxidative stress, and calcium overload specifically trigger mitochondrial dysfunction.

On the other hand, relevant research data show that the concentration of free Ca^2+^ in mammalian cells is generally maintained at 100–200 nmol/L. In contrast, the concentration level of Ca^2+^ in extracellular and organelle reaches mmol/L [89]. Therefore, endogenous calcicoptosis may become a new research focus. As the most critical intracellular calcium pool, ER is widely distributed in neurons [90]. Store-operated calcium entry (SOCE) mediated by internal calcium storage can be induced by subtle changes in ER Ca^2+^, which trigger calcium influx into the cytoplasm, thus affecting various processes such as cell differentiation, maturation, and apoptosis [91]. Bu’s team ingeniously constructed a ZIF-82 nano-system loaded with calcium pump inhibitor berberine (BER) on the outer surface of UCNP. ZIF-82 was activated by up-converting UV light and the acidic tumor microenvironment to release nitric oxide (NO) and BER molecules. Then, the synergistic action of the two leads to intracellular calcium overload and induces cell calcicoptosis [92]. This strategy converts endogenous non-toxic Ca^2+^ into toxic molecular weapons to kill tumor cells for the first time, providing guidance and reference for further developing the “self-destructive” anti-tumor strategy. Through bioinformatics analysis, Professor Hu's research team found that the ER calcium channel protein TMCO1 is crucial for cells to cope with ER calcium overload and maintain calcium homeostasis. Studies found that inhibiting the expression of TMCO1 would lead to intracellular Ca^2+^ imbalance, thus effectively blocking the malignant proliferation of tumor cells [93]. This study extends the scientific view that calcium homeostasis is involved in forming malignant phenotypes in tumors and provides an important target for cancer treatment strategies through calcium imbalance.

It is noteworthy that mitochondria also play a critical multi-functional role in cells, including the generation of adenosine triphosphate (ATP) [94], regulation of redox [95], maintenance of calcium homeostasis [96], and transmission of metabolic signals [97], indicating that mitochondria may be another ideal target for inducing endogenous calcium death. Bao’s team reported a novel MOFs-based core–shell nano-agent that achieves synergistic anti-tumor therapy of dual mitochondrial damage through oxidative stress and calcium overload triggered by near-infrared light [98]. Under near-infrared light, the intracellular acidic environment and oxidative stress induced by upconversion nanoparticles can cause a large amount of calcium influx, resulting in mitochondrial calcium overload. The results showed mitochondria induced oxidative stress in tumor cells, thereby achieving intracellular endogenous calcicoptosis. This study efficiently kills tumor cells by destroying the synergistic mechanism of mitochondria, which brings a new idea for tumor endogenous calcium overload.

Calcicoptosis mainly utilizes the retention and accumulation of calcium ions in tumor cells to cause persistent calcium overload, which interferes with or hinders tumor cell metabolism and proliferation, resulting in the cell malfunctioning and eventually inducing cell death [85]. Calcicoptosis has been applied to some calcium treatment cases [99, 100]. However, the more specific mechanism remains to be further explored, and how to deliver calcium ions more efficiently and selectively to achieve a calcium overload state should be considered.

### Cuproptosis

As a cofactor of various essential enzymes, copper ions are essential in organisms, and their combination with enzymes can assist in blood clotting, hormone maturation, and cell processing [101, 102]. Studies on copper ions have focused more on biocatalytic activity [103–105] for a long time. The TEM has many characteristics different from typical cell environments in cancer, such as low pH value, hypoxia, H_2_O_2_, and GSH overexpression [106, 107]. Therefore, under the acidic condition of TME, copper ions can realize the valence transfer by taking advantage of the reducibility of GSH and the oxidability of H_2_O_2_, thus completing oxidative stress and effectively triggering the cytotoxicity of highly toxic ·OH [108, 109].

Unlike biocatalysis, studies have found that when Cu^2+^ homeostasis is broken in the human body, the copper overload will lead to cell death and injury [102], but the related mechanism needs further exploration. According to the latest research report, Peter Tsvetkov and Todd R. Golub’s team have revealed a novel mode of cell death called cuproptosis and demonstrated that copper-induced cell death is mediated by protein lipidation [110].

Due to the severe protein toxic pressure in tumor cells, such as increased protein turnover and genomic instability, the balance between protein production and degradation is destroyed, and protease activity is inhibited [111–113]. Copper chelation is an effective treatment in treating genetic diseases with dynamic copper balance [114]. Early studies have found that Elesclomol is a highly fat-soluble Cu^2+^ binding molecule that can transport Cu^2+^ across membranes, restore the mitochondrial function of copper-deficient tissues, and activate the generation of related proteases [115–117]. Guthrie has provided convincing evidence that Elesclomol small molecules can carry copper across complex biological barriers and improve tissue copper metabolism. Elesclomol can also target copper operators and transport them to different tissues [115, 118], revealing the possibility to be applied to various cancer types and providing a new starting point for developing copper ionophores. Besides, it is worth noting that Elesclomol was found to transport copper to mitochondria and induce cell death in previous studies [119–121]. This finding deserves further discussion.

The exploration of copper toxicity by Todd R. Golub’s group began in 2019. Golub demonstrated using a functional genomics approach that proteasome inhibitor resistance to tumor cells is associated with a shift in mitochondrial energy metabolism, revealing how Elesclomol promotes copper-dependent cell death [122]. The team delved deeper into copper toxicity to clarify how copper dependence leads to cell death. They tested the cell-killing effects of 1448 copper-laden drugs in 489 cell lines and found that all of them worked. To further test the effect of copper on cells, they treated cells with buthionine sulfoximine (BSO, depletion of intracellular copper chelating agent GSH) in combination with Elesclomol. They found that cell death was induced, and Tetrathiomolybdate (TTM, copper chelation) combined with Elesclomol did not affect cell growth. These results suggest that cell death induced by copper ionophores mainly depends on intracellular copper accumulation. However, improving the copper-binding ability of these compounds results in a loss of cell killing ability, and copper chelation eliminates the toxicity [122]. In addition, compared with known cell death modes such as apoptosis [123, 124], necrotic apoptosis [125, 126], pyroptosis [127], and ferroptosis [22], cell death induced by copper does not involve known pathways, which proves that cell death induced by copper ionophores has a unique approach.

In order to explore the regulatory mechanisms associated with copper-induced cell death, Todd Golub and his team conducted experiments. They found that mitochondrial respiration involved cell death, and ATP was less affected by glycolysis, suggesting that cuproptosis was mediated by mitochondrial respiration. According to metabolomics studies, significantly more TCA-related metabolites were found in copper-sensitive cells, which further confirms that Cu^2+^ does not directly participate in the electron transport chain (ETC) and only plays a role in the tricarboxylic acid (TCA) cycle. These results suggested that mitochondrial respiration was necessary for cuproptosis and that components of the TCA cycle were essential targets of this cell death pattern.

Meanwhile, to identify the specific metabolic pathway that mediated copper toxicity, Todd Golub's team used multiple CRISPR knockout screening to identify a critical gene that promotes cuproptosis, ferric reductase 1 (FDX1). The reductase encoded by this gene could convert Cu^2+^ into more toxic Cu^+^ and provide direct Elesclomol targets [122]. In addition, they found that cuproptosis was highly consistent with protein lipoylation mediated by mitochondrial metabolism. It was further confirmed that FDX1 and protein lipidation are the critical regulators of cuproptosis, and FDX1 was the upstream regulator of protein lipidation.

**Fig. 4:**
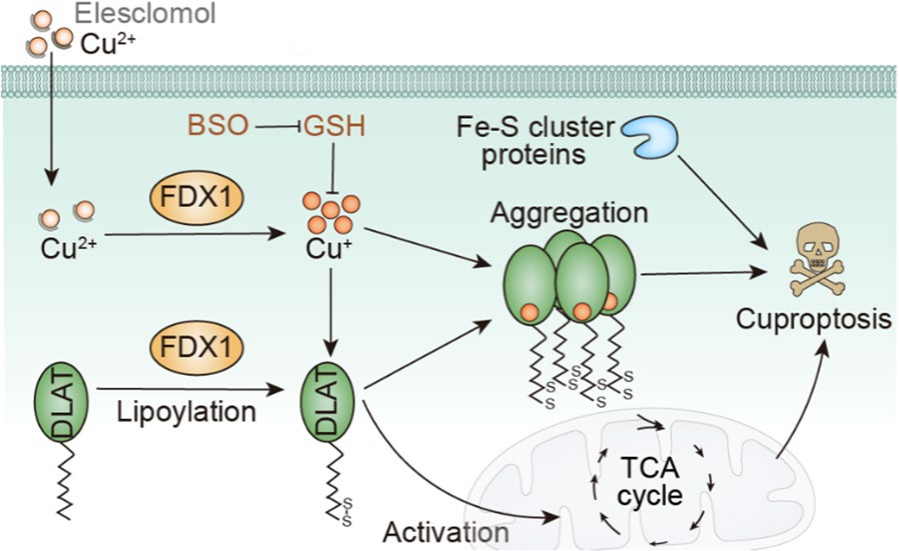
Schematic illustration indicates the mechanism of cuproptosis induction [110]

Finally, to explore the link between copper toxicity and protein lipoylation, Todd Golub's team hypothesized that copper might directly bind to the lipoylated proteins. They demonstrated that the thiooctyl portion of the protein was required for copper binding. Copper binds directly and induces oligomerization of dihydrolipoamide s-acetyltransferase (DLAT). In addition, copper interferes with iron-sulfur (Fe-S) clusters, components of several key metabolic enzymes. These results suggest that the cellular effects of copper overload were the same as that of copper ionophores. Excessive copper promotes the aggregation of lipoylated proteins and the instability of Fe-S cluster proteins, ultimately leading to toxic protein stress and cell death (Fig. 4).

Part of the mechanism of cuproptosis has been confirmed to occur in bacteria and yeast. Therefore, this study could shed light on various biological processes, including the antimicrobial properties of microorganisms producing copper ionophores [128]. Moreover, the genetic diseases associated with copper disorders include Wilson's [129]. In addition, based on the cuproptosis properties, copper ionophores may help treat tumors that rely on mitochondria to produce energy. Copper ionophores are expected to be developed to treat cancers susceptible to cuproptosis, such as those expressing FDX1.

Much different copper ionophores induce cell death by the cuproptosis mechanism, which depends entirely on copper ions' availability [110]. Copper ionophores usually form neutral lipophilic complexes with copper, promoting the copper accumulation of intracellular copper [130]. The stability of the copper ionophores determines whether the Cu^2+^ is retained or not [131]. When the copper ionophores are too stable, the release capacity of the Cu^2+^ is small, and its availability is low. Similarly, too low stability will lead to competitive chelation of Cu^2+^ by other organisms. Only copper ionophores with moderate affinity for Cu^2+^ are most advantageous, and free ligands bind to copper at high concentrations and release at low concentrations. At the same time, the release of Cu^2+^ may be related to the protonation of copper ionophores. When copper ionophores are in an acidic environment or enter the lysosomes of cells, protons will bind to them, releasing Cu^2+^ [130]. In addition, lipophilicity and redox potential also affect the activity of copper ionophores. Copper ionophores bond with Cu^2+^ outside the cell and then passively diffuses into the cell through the phospholipid bilayer. If the copper ionophores are too hydrophilic, they cannot effectively cross the cell membrane [132]. When Cu^2+^ is reduced to a lower state, the copper ionophores lose copper due to weakened affinity [133].

In general, it has been demonstrated that cuproptosis possesses its exclusive mechanism, which is undoubtedly worth expecting. Different from existing approaches, cuproptosis is caused by the abnormal aggregation of lipoacylated proteins and the loss of iron-sulfur cluster proteins. Binding copper ions to the lipoacylated components in the tricarboxylic acid cycle triggers a toxic stress response to proteins, ultimately leading to cell death [134]. Now many copper ionophores have been developed (Table.2). With the development of nanotechnology, exploiting nano-formulations with the cuproptosis mechanism to increase the intracellular copper content through its delivery ability is expected to achieve specific damage to tumor cells. Combining cuproptosis and material science may stimulate a new class of efficient anti-tumor strategies.

**Table 2:** Classification of copper ionophores

Classification	Abbr	Full name	References
Elesclomol	ES	Nʹ1,Nʹ3-dimethyl-Nʹ1,Nʹ3-bis(phenylcarbonothioyl)propanedihydrazide	[115, 135]
Dithiocar-bamates (DTCs)	PDTC	pyrrolidine dithiocarbamate	[136]
	DEDTC	Diethyldithiocarbamate	[137]
	DSF	disulfiram	[138]
	GGTDTC	DEDTC prodrug (GGTDTC) activated by c-glutamyltransferase (GGT)	[139, 140]
	DPy	2,2ʹ-dithiodipyridine	[141]
Thiosemicarbazones (TSCs)	GTS	glyoxal-bis(thiosemicarbazone)	[142]
	GTSM	glyoxal-bis(4-methylthiosemicarbazone)	[143]
	ATSM	diacetyl-bis(N4-methylthiosemicarbazone)	[144]
	DpT	di-2-pyridylketone thiosemicarbazone	[145]
	Dp44mT	Di-2-pyridylketone-4,4,-dimethyl-3-thiosemicarbazone	[146]
	DpC	di-2-pyridylketone 4-methyl-4-cyclohexyl-3-thiosemicarbazone	[147]
Hydroxyquinolines (HQs)	CQ	7-iodo- 5-chloro-8-hydroxyquinoline	[148]
	8-OHQ	8-hydroxyquinoline	[149]
	AMHQ	5-aminomethyl-8-hydroxyquinoline	[150]
	NQ	5-chloro-8-hydroxyquinoline and nitroxoline	[151]
	PBT2	5,7-dichloro-2-[(dimethylamino)methyl]-8-hydroxyquinoline	[152, 153]
	GluHQs	β-glucoconjugates of HQs	[154]
	GalHQs	galactoconjugates of HQs	[155]
Hydroxyflavones (HFs)	3-HF	3-hydroxyflavone	[156]
	PHF	2,4-dinitrobenzenesulfonate conjugated to 3-HF	[157, 158]
	PL-I	β-diketo analog of piperlongumine	[159]
Curcumin	Curc	1,7-bis(4-hydroxy-3-methoxyphenyl)-1,6-heptadiene-3,5-dione	[160]

### Other approaches to inducing tumor cell death by metal ions

In addition to the above new modes of cell death, more metal ions have realized the anti-cancer application through different approaches such as interfering with osmotic pressure, triggering biocatalysis, activating immune pathways, and generating the prooxidant effect, which greatly complements the means of cancer therapy.

#### Interference with osmotic pressure

The relatively stable osmotic pressure of the intracellular and extracellular fluid is the condition for maintaining cell morphological function. Na^+^ and K^+^ play an essential role in maintaining cell osmotic pressure homeostasis. Na^+^ is the most critical cation affecting the extracellular fluid, and the osmotic pressure of intracellular fluid almost depends on the concentration of K^+^ [161]. However, once concentration gradients of these ions are interfered with, beyond the scope of automatic regulation of cells, changes in osmotic pressure may lead to cytoskeleton destruction, cell cycle stagnation, and, ultimately, cell death [162]. Therefore, exogenous delivery of metal ions to interfere with the osmotic pressure of tumor cells to induce cell death is expected to be a feasible strategy without excessive systemic toxicity. Li et al. [163] cleverly delivered NaCl nano-crystals wrapped in SSSS-VHMS to the tumor cells. Excessive GSH in tumor cells would trigger the rapid degradation of SSSS-VHMs, significantly consume GSH and explosively release Na^+^ and Cl^−^. They were causing a surge in osmotic pressure that synergistically induces cell death. Ding et al. [163] have prepared K_3_ZrF_7_:Yb/Er upconversion nanoparticles (zrNPs). zrNPs are similar to ion banks, which release abundant K^+^ and [ZrF_7_]^3−^ ions when dissolved in the interior of cancer cells, resulting in a surge of intracellular osmotic pressure and destruction of cellular environmental homeostasis and promoting tumor cells’ lysis. Therefore, the strategy of interfering with cell homeostasis through the targeted delivery of these metal ions, which determine the osmotic pressure of cells, also provides a new idea for cancer therapy.

#### Activation of biocatalysis

The high efficiency and specificity of biocatalysis undoubtedly make it have the potential to be applied in the medical field [164]. In cancer, biological enzymes such as glucose oxidase and adenosine triphosphate bisphosphatase have been applied [165]. However, due to the high synthesis cost of biological enzymes and the unique nature of the tumor microenvironment, the clinical promotion of biological enzymes is greatly limited [166]. Therefore, Nanozymes have attracted extensive research interest due to their close connection between nanotechnology and biology [165]. Some metal ions with catalytic functions were designed as Nanozymes. They transform H_2_O_2_ overexpressed in TME into poisonous ·OH, thus causing irreversible oxidative damage to tumor cells. This strategy has used the excellent oxidation–reduction properties of metal ions and dramatically reduced the application cost of biological enzymes. In addition to iron and copper ions mentioned above, many metal ions have also been found to activate biocatalytic reactions. Jiang et al. [167] designed a new TME-responsive nano-platform Co/ZIF-8/ICG/Pt (CZIP). The indocyanine green (ICG) could generate singlet oxygen and promote apoptosis of cancer cells under near-infrared irradiation. The doping of Co^2+^ would catalyze H_2_O_2_ overexpressed in TME to generate ·OH, killing tumor cells. Experimental results also showed that the two approaches had a synergistic anti-tumor effect. Dong et al. [168] developed multi-functional nanozymes by coating dendritic SiO_2_ on homogeneous Bi_2_S_3_ nanorods and loading ultra-small CeO_2_ into the mesopore. Under acidic conditions, it showed the dual catalytic activity of oxidase and catalase, which improved the degree of oxidative stress in TME, thus significantly enhancing the treatment efficiency of ROS-mediated therapy. Besides, many transition metal ions also have homogeneous catalytic activity such as Mn^2+^ [169], Ti^3+^ [170], Ag^+^ [171], and Mo^4+^ [172]. This approach of killing tumor cells by activating biocatalysis has produced good therapeutic effects in vitro and in vivo, potentially complementing existing cancer therapies.

#### Activating the immune pathway

Immunotherapy is also a cancer therapy that has developed continuously in recent years. It utilizes the immune mechanism in the human body to fight tumor cells and is an integral part of future cancer research strategies [173]. However, the limited immune response and immunosuppressive characteristics of the TME hinder its development [174, 175]. In the latest studies, metal ions have been found to play an essential regulatory role in immunotherapy. The introduction of metal ions can enhance the immune response and activate the immune pathways in tumor cells to play an anti-tumor effect [176]. Dai's team constructed a novel metal-polyphenol network (DSPM) using polyphenol derivatives and metal ions coordination [176]. In acidic TME, Mn^2+^ is released into the cytoplasm by the disintegration of DSPM, which could activate the stimulator of interferon genes (STING) pathway, promoting dendritic cells' maturation. Furthermore, tumor-specific antigen was presented to T cells to enhance anti-tumor immunity. Besides, tumor immune responses are also assisted by Mg^2+^. In the study of Lotscher's team, Mg^2+^ might target LFA-1 (integrin, composed of CD11a and CD18) and initiate downstream signaling pathways by binding to metal ion-dependent adhesion sites on CD11a and CD18 to promote T cell degranulation and kill target cells [177]. In addition, Ni^2+^ [178], Ca^2+^ [179], Zn^2+^ [180], and other metal ions are also involved in cellular immune response, regulation, signal transduction, and etc. However, activating immune pathways by metal ions is a novel strategy, which inevitably faces many challenges in studying related mechanisms. However, there is no denying that this strategy has paramount theoretical significance and practical value. It breaks through the barrier of immunology and other fields, providing many new ideas for basic immunity research and clinical application.

#### Generating the prooxidant effect

Many anti-cancer drugs have been found to target copper ions within TME, generating a prooxidant effect, leading to ROS generation and apoptosis induction. Copper ion levels are considerably elevated in almost all cancers [181]. Therefore, some research groups have focused their work on the complexes with endogenous copper ions for new anti-cancer drugs with lower toxic effects [182, 183]. Mohd Farhan's group [181] believed that such a mechanism explains the anti-cancer effect of epicatechin-3-gallate and its preferential cytotoxicity toward cancer cells. Due to the redox activity of copper complexes, they can present a dual role. The compounds with both antioxidant and prooxidant activities could act as protective molecules that dismutate superoxide radicals and function as anti-tumor species since they can produce ROS, which could damage different biomolecules and induce cellular senescence and death. Nicolas Veiga's team studied a series of homoleptic copper (II) complexes with amino acids and dipeptides [184]. The antioxidant behavior of complexes was compared by measuring the superoxide dismutase (SOD)-like activity, and the prooxidant activity was performed by assessing the oxidative damage to 2-deoxy-D-ribose (using the TBARS method). Their findings showed that Cu-amino acid complexes were strong ROS producers and moderate SOD mimics. Conversely, Cu-dipeptide-phen complexes were good SOD mimics but poor ROS producers [185]. Therefore, their intrinsic reactivity needs to be evaluated when selecting coordination compounds.

Interestingly, vitamin C (VC) is a water-soluble antioxidant [186], but it can switch to a prooxidant to generate ROS via its autoxidation catalyzed by copper and iron ions. Du’s study [187] explained that VC and its two-electron oxidative product (DHA) construct an efficient redox cycle with the aid of intracellular glutathione and copper ions, thereby facilitating ROS generation, which showed that VC might target the TME and possess the anti-tumor ability. Some chemotherapy drugs can also target the iron ions and induce the prooxidant effect [188–191].

Besides, many metal ions have gradually induced tumor cell death through different pathways. Such as Pt^2+^ and its complexes can target tumor cell DNA, block transcription and replication, and thus start apoptosis [192]. However, severe side effects and increased drug resistance have limited its clinical application [193]. Studies have also confirmed that Ir^3+^ and Re^+^ can induce apoptosis and autophagy and even show higher tumor cytotoxicity than cisplatin [194]. Complexes based on Ru^2+^ and Os^2+^ can often inhibit the cell cycle and trigger apoptosis mediated by cell cycle arrest, showing high anti-tumor activity [195]. More and more new anti-tumor approaches based on metal ions have been discovered (Table.3), and it is believed that metal ions will be widely used against cancer.

**Table 3:** Approaches and examples of inducing tumor cell death by metal ions

Metal ions	Nano-platform example	Inducing approach	Main principle	References
Fe^2+^, Fe^3+^	RCH	Ferroptosis	Lipid peroxides accumulation	[196]
	NMIL-100@GOx@C	Apoptosis	Biocatalysis	[197]
Cu^+^, Cu^2+^	Cu-Elesclomol	Cuproptosis	Protein toxic stress	[110]
	DSF@PVP/Cu-HMPB	Apoptosis	Biocatalysis	[198]
Ca^2+^	SH-CaO_2_	Calcicoptosis	Calcium overload	[85]
Na^+^	Na_2_S_2_O_8_	Pyroptosis	Osmotic pressure interference	[199]
K^+^	K_3_ZrF_7_:Yb/Er	Pyroptosis		[164]
Co^2+^	Co/ZIF-8/ICG/Pt	Apoptosis	Biocatalysis	[167]
Ce^2+^, Ce^3+^	PEG/Ce-Bi@DMSN	Apoptosis	Biocatalysis	[168]
Mn^2+^	MS@MnO_2_	Apoptosis	Biocatalysis	[200]
	DSPM	Apoptosis	Immune activation	[176]
Ag^+^	AgNC–GOx	Apoptosis and autophagy	Biocatalysis	[201]
Ti^3+^	D-MOF (Ti)	Apoptosis	Biocatalysis	[170]
Mo^4+^	MoP_2_	Apoptosis	Biocatalysis	[172]
Zn^2+^	HZ@GD	Apoptosis	Glycolysis inhibition	[202]
Mg^2+^	DNF	Apoptosis	Promoting DNA crosslink	[203]
	Mg^2+^	Apoptosis	Immune activation	[177]
Ni^2+^	mNiO	Apoptosis	Biocatalysis	[204]
	[Ni(La1)_2_(Lb2)]·CH_3_OH	Apoptosis and autophagy	Mitophagy	[205]
Re^+^	CA-Re	Pyroptosis	Immune activation	[206]
Pt^2+^	Pt(II) complexes (C1–C6)	Apoptosis and autophagy	Impeding DNA synthesis	[192]
Ir^3+^	Ir(III)-Re(I)	Apoptosis and autophagy	Cell cycle inhibition	[194]
Ru^2+^	[Ru(bpy)_2_(phcpip)] (ClO_4_)_2_	Apoptosis	Cell cycle inhibition	[207]
Os^2+^	[Os(η6-pcym)(bphen)(dca)]PF6	Necroptosis	Eliminating cancer stem cell (CSC)	[208]

## Metal ions mediate cancer diagnosis and new nano-theranostics platform

### Bioimaging technology guided by metal ions

**Fig. 5:**
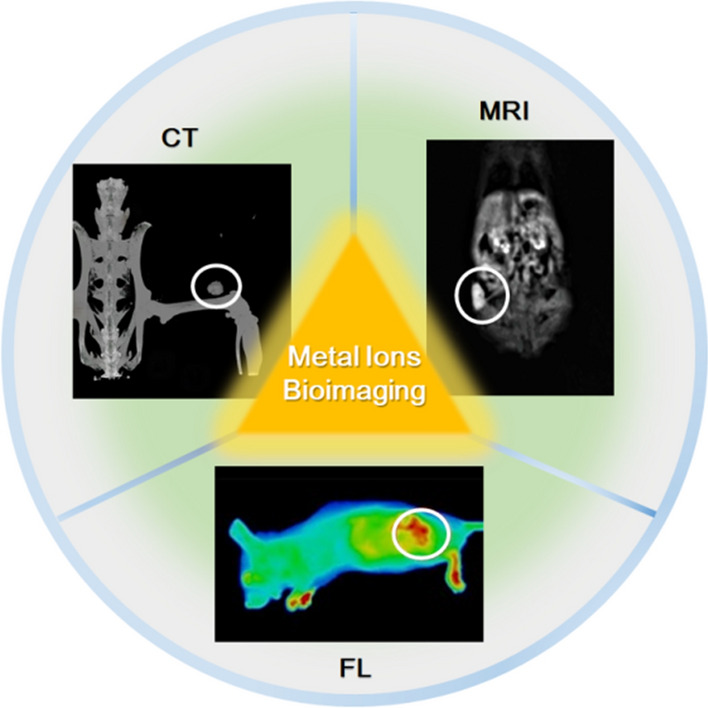
Typical diagrams of bioimaging technology guided by metal ions

The process of clinical cancer diagnosis mainly involves imaging examination, preliminary diagnosis, pathological diagnosis, and further diagnosis. The imaging examination can intuitively show the tumor’s location and size through various bioimaging technologies, which have played a pivotal role in cancer diagnosis and treatment [209, 210]. Currently, the bioimaging technologies applied in cancer mainly include magnetic resonance imaging (MRI), X-ray computed tomography (CT), ultrasound imaging (including color Doppler ultrasound), positron emission tomography (PET), photoacoustic imaging (PAI), and fluorescence imaging (FL) [211, 212]. Metal ions mainly work for MRI, CT, and fluorescence imaging (Fig. 5).

#### MRI

Magnetic resonance imaging (MRI) is realized by the effect of an external high-frequency magnetic field and the signal generated by the radiation energy from the internal material to the surrounding environment. To be precise, the combinations of time-varying gradient magnetic fields and pulse sequences of radiofrequency waves provide the spatial distribution of signals emitted from protons, displayed as high-resolution and multidimensional images [213]. In recent years, MRI is often used in brain tumor detection because it causes less radioactive or biological damage to the body [214]. With the improvement of MRI resolution, the technique shows the utility of increasing the extent of resection for tumors and preventing injury to important demic structures while incorporating technologies such as intraoperative monitoring [215].

Over the past few decades, Gd-based contrast agents have dominated the MRI market in clinical applications. They can reduce the longitudinal relaxation time of nearby protons, just like the T_1_-weighted contrast agent [216–218]. Nevertheless, they are controversial for being nephrotoxic, and the FDA has issued a general warning concerning their use [219]. Therefore, people have been actively looking for substitutes, strongly prompting the development of paramagnetic metal ions such as Fe^3+^ and Mn^2+^ for MRI [220]. Surprisingly, using the agents that provide contrast according to the chemical exchange saturation transfer (CEST), such as paramagnetic Ln^3+^, can increase the chemical shift difference between two proton pools [221, 222]. In addition, transition metal ions [223–225] or lanthanides can also accelerate the relaxation time, thus speeding up the imaging acquisition time [226–228].

Ni et al. [229] presented the first example of T_2_-weighted imaging in an upconversion nanoparticles sensitizer (Yb^3+^) and activator (Ho^3+^), and it can also be used in UCL and CT imaging due to the high X-ray attenuation properties and magnetic properties of Ho^3+^. Hence, the system has achieved high-performance multi-modal MRI/UCL/CT imaging in a single upconversion nanoparticle [230], which facilitates the accurate diagnosis of brain tumors in the preliminary stage. Meanwhile, Zeng’s team [231] synthesized a new kind of hybrid lanthanide nanoparticles by doping with 0%, 50%, or 100% Ln^3+^ (Ln^3+^ = Yb^3+^, Er^3+^, or Dy^3+^), which can be used as T1/T2 dual-weighted MRI contrast agent and mediate tumor diagnosis synergistically. The experiment showed that the r_2_/r_1_ value of BaGdF_5_ NPs can be readily adjusted from 2.8 to 334.8, and the contrast effect was excellent when the BaGdF_5_ nanoparticles were doped with 50% Er^3+^. These findings have provided a simple and universal method for designing simultaneous T_1_/T_2_-weighted enhancers for cancer diagnosis.

#### CT imaging

X-ray computed tomography (CT) imaging is one of the most widely used imaging techniques in medical diagnosis, which utilizes computer-processed X-rays to produce tomographic images [213]. It has numerous advantages of considerable tissue penetration depth, high spatial resolution, and fast scanning speed [232, 233]. Accurate volume imaging of the whole body or individual organs can achieve submillimeter isotropic resolution in 5 to 20 s [233]. On X-ray examination, hard tissue (bone and cartilage) is easily distinguishable from surrounding soft tissues. However, the contrast difference between different soft tissues is sometimes so slight that they need a transient contrast-enhancing X-ray contrast agent (CAs) to obtain more detailed images.

Since the 1970s, CT has been a major medical breakthrough, including tumor diagnosis. Small iodinated molecules such as CT contrast agents are commonly used in the clinic. However, they are unsuitable for detecting TME due to their nontargeting, short half-life, and side effects on the human body [234]. Hence, some metal ions and their compounds are designed for CAs in CT imaging, such as Bi^3+^ [235] and W^4+^ [236, 237], because they possess a high X-ray absorption coefficient and atomic number. Moreover, the photoelectron effect leads to contrast enhancement, so the CAs based on metal ions can work well in conjunction with cancer phototherapy [238]. Yang's group [239] designed AR-Bi@SiO_2_-Gd/DOX nanoparticles (NPs), which were developed by integrating gadolinium complex within doxorubicin (DOX)-loaded protective silica shell as well as bismuth nano-core. The NPs were found to have a very long retention half-life period of 104.5 h in the tumor. The experiment showed that the NPs possessed excellent CT/MRI effectiveness. Undoubtedly, prolonged retention has been more conducive to anti-cancer applications. Wang's team [240] developed BiVO_4_/Fe3O_4_@PDA NPs that had CT/MRI/PA multi-modal imaging effect and realized the collaborative treatment of radiotherapy (RT) and photothermal therapy (PTT) in oral epithelial carcinoma. Cheng et al. [241] synthesized biodegradable FeWO_X_-PEG-RGD NP_S_. The system implemented phototherapy guided by CT/MRI dual-mode bioimaging. With high imaging accuracy and superb anti-cancer efficiency, this report also revealed the prospect of tungsten ions as CT imaging contrast agents.

#### Fluorescence imaging

The intensity of fluorescence signal emitted by ground state fluorescent substance after excitation is linear with the amount of fluorescein in a particular range, which is the theoretical basis for applying fluorescence imaging system in biological research [242]. In recent years, owing to the advances in reducing photon scattering, promoting light absorption, and autofluorescence through innovations in the broad 700–1700 nm NIR window, NIR fluorescence affords high imaging resolution with increasing tissue penetration depths [243]. Therefore, the NIR fluorescence imaging has quickly gained more attention in cancer diagnosis. It can track and observe diseases at the molecular level and evaluate tumor development and metastasis through real-time wide-field imaging of target cells and gene expression, thus guiding cancer treatment [244].

Metal quantum dots have attracted much focus for fluorescence imaging due to their high quantum yield, high molar extinction coefficient, efficient Stokes shift, high resistance to photobleaching, and exceptional resistance to chemical degradation [245]. Quantum dots absorb light and generate excitons in nano-crystals, electron–hole complex induced luminescence [246]. Quantum dots based on heavy metal elements, such as Hg and Pb, have been extensively studied in the past, but their internal toxicity limits their biological applications. Therefore, many researchers are committed to the synthesis of quantum dots without heavy metal components such as MnO_2_ QDs [247], InP QDs [248], and AgS QDs [249] et al. to improve biocompatibility.

According to new research, doping transition metal ions or lanthanide metal ions in quantum dots (QDs) typically introduces dopant-related energy levels within the intermediate gaps of the subject and can dramatically alter existing properties or even generate new functions [250–255]. Zhang et al. [256] studied the successful preparation of Mn^2+^, Cu^2+^, and Ni^2+^-doped CdS quantum dots. On the premise of good water solubility, they were stable and non-toxic to be directly used for bioimaging. X-ray absorption fine structure (XAFS) analysis proved that dopants were inside the quantum dots rather than on the surface, which can effectively overcome the "self-purification" effect. Wang's team [257] designed Zn^2+^–cryptolepine–cyclen complexes with dual fluorescence characteristics. The selective fluorescence imaging of the nucleus and mitochondria of A549R cancer cells was conducted with a promising in vivo safety profile. Lv's group [258] has offered a strategy to synthesize YVO_4_:Nd^3+^-HMME@MnO_2_-LF NPs (YHM). The YVO_4_:Nd^3+^ core exhibited good NIR-II fluorescence properties, enabling YHM to act as promising probes for fluorescence imaging of vessels and gliomas. The system has been successfully applied in active tumor-targeted imaging and treatment in vitro and in vivo, which provided insights into exploring the theranostic agents based on metal ions-doped nanoparticles.

### New nano-theranostics platform based on metal ions

“Theranostics” is a term coined by combining the words “therapeutics” and “diagnostics” [259]. Over the years, the theranostics platform, or the marrying of therapy and diagnosis, has increasingly been employing nano-based approaches to anti-cancer applications. [19, 260]. Therefore, this nano-platform can monitor the therapeutic effect through the size, location, morphology, and other information obtained from cancer diagnosis, realizing the visualization of the therapeutic process as a result of advances in nanotechnology [261], which is of great significance for developing treatment protocols, guiding drug dosage, and assessing prognosis [262].

Traditional theranostics platforms have been designed by combining anti-tumor drugs with bioimaging technology, which suffers many limitations, such as uncontrolled cargo release, insufficient tumor deposition, long-term toxicities, and potential mutual inhibition between components [263, 264]. Aiming at these problems, researchers perceive that the well-designed nano-platform should be able to respond to endogenous or exogenous stimuli to increase the drug targeting efficacy, reduce side effects and toxicities of payloads, and achieve multifunction. The endogenous triggers include pH value, hormone level, enzyme concentration, small bio-molecules, glucose, or redox gradient, which are related to the pathological characteristics of cancer. Meanwhile, the exogenous triggers usually contain temperature, light, magnetic field, ultrasound, electric pulse, and high energy radiation [265–267].

Therefore, the new nano-theranostics platform based on metal ions has gradually developed with the continuous progress of nanotechnology. As mentioned above, metal ions can induce tumor cell death and simultaneously mediate cancer diagnosis, which benefits nano-systems multifunction. Moreover, metal ions widely exist in organisms, which avoids severe toxic and side effects. Surprisingly, various metal ions have been found to respond to the endogenous stimulus in TME, such as the pH value, H_2_O_2_ concentration, GSH expression, glucose content, and expression of enzymes. These endogenous signals can serve as targets of metal ions to achieve responsive release and in-situ killing of tumor cells. Meanwhile, many metal ions can absorb exogenous energy from light, radiation, ultrasound, and electricity, which contributes to realizing the controlled release of nano-system and inducing synergetic therapy such as photothermal therapy (PTT), photodynamic therapy (PDT), and sonodynamic therapy (SDT).

Based on the above, Gao’s group synthesized a novel nanoprobe consisting of upconversion luminescence (UCL) nanoparticles as a core and a coordinatively unsaturated Fe (III)/gallic acid complex as a shell. After intravenous injection, the nanoprobes bind to transferrin, enhancing tumor targeting through damaged blood vessels and thus accumulating in the tumor area. Once the tumor absorbs the probe via transferrin receptors, the low pH of the tumor microenvironment would activate an unsaturated coordination shell, enabling the T_1_ effect of Fe (III) by breaking the superexchange coupling within the unsaturated coordination complex. The released Fe (III) can also accelerate tumor cell death by upregulating ROS, and the residual Ga-Fe (III) on the probe surface acts as a healing center for laser irradiation of PTT [268]. Besides, our group developed GBD-Fe, a nano-formulation that effectively integrated chemotherapy (CT), chemodynamic therapy (CDT), and photothermal therapy (PTT). GBD-Fe used gold nanorods as photothermal agents and encapsulated doxorubicin to amplify Fe (III)-guided CDT effects by producing H_2_O_2_ and reducing the intracellular glutathione levels. Fe (III) enhanced the T_1_-weighted image of MRI. In vitro and in vivo experiments demonstrated this tri-pronged therapy's enhanced accumulation and anti-tumor effects under magnetic resonance imaging (MRI) guidance. This tri-pronged CT/CDT/PTT approach effectively induced tumor cytotoxicity and inhibited tumor growth in tumor-bearing mice, representing a promising strategy for treating tumors effectively [269]. Some typical examples of new nano-theranostics platforms based on metal ions are enumerated in Table.4, and we do hope that there will be more feasible schemes to provide new opportunities for people to conquer cancer.

**Table 4:** New nano-theranostics platforms based on metal ions and their compounds

Nano-platform	Involving therapy	Involving diagnosis	References
UCNP@GA-Fe(III)	Fe^3+^ (CDT)	Fe^3+^ (MRI)	[268, 270]
GBD-Fe	Fe^3+^ (CDT)	Fe^3+^ (MRI)	[269]
DOX@Mn-Alg	Mn^2+^ (CDT)	Mn^2+^ (MRI)	[271, 272]
pCo_3_O_4_	Co_3_O_4_ (PTT)	Co^2+^ (MRI)	[273]
ipGdIO-Dox	Fe^2+^ (Ferroptosis)	Fe^2+^, Gd^3+^ (MRI)	[274]
CuS-NiS_2_	CuS-NiS_2_ (PTT/PDT)	Cu^2+^, Ni^2+^ (MRI)	[275]
MPDA-WS_2_@MnO_2_	WS_2_ (PTT)	W^4+^(CT), Mn^2+^ (MRI)	[276]
FeWO_X_-PEG-RGD	FeWO_X_(PTT/PDT/CDT)	W^4+^(CT), Fe^3+^ (MRI)	[241]
PEI-Bi_2_Se_3_	Bi_2_Se_3_ (PTT)	Bi^3+^ (CT)	[277]
Bi-Ag@PVP	Bi-Ag (PTT)	Bi^3+^ (CT)	[278]
Bi_2_S_3_@Ce6-CeO_2_	Bi_2_S_3_ (PTT)	Bi^3+^ (CT)	[279]
CdTeSe/ZnS (QDs)	CdTeSe/ZnS (PTT)	CdTeSe/ZnS QDs (FL)	[280]
CCM@AT	Ag_2_S (PTT)	Ag_2_S QDs (FL)	[281]

## Summary and prospect

Undoubtedly, the new anti-cancer explorations based on metal ions have been significant due to their unification of theory and practice. Inducing tumor cell death by metal ions has continuously achieved exciting results. More contrast agents based on metal ions have gradually become the critical components in cancer diagnosis. Metal ions have gained more extensive anti-cancer applications with the advancement of nanotechnology.

Compared with traditional therapy, metal ions treatment can kill tumor cells with fewer side effects on normal tissues and organs. The excellent responsiveness of metal ions to TME enhances the accumulation of nano drugs in tumors, which increases the targeting efficiency and achieve specific treatments for different cancer types. At the same time, external energy can realize the controlled release of nano-drugs based on metal ions and mediate synergistic treatment. Besides, it has been confirmed that most tumor cells are not resistant to metal ions [282], which enables metal ions to apply to multi-course treatment. Further, the metal ion-mediated cancer diagnosis is conducive to monitoring and controlling the therapeutic process, improving treatment efficiency.

Nevertheless, many problems need to be analyzed and reflected before practical clinical applications. In the macro view, how to further improve the efficiency of targeted delivery of metal ions and promote their accumulation in tumors is inevitably a vital problem, which may depend on the more sophisticated design of the structure and function of nanomaterials. Meanwhile, metal ions-mediated single therapy may face the problem of insufficient efficacy, and single-mode imaging may also bring false positives and reduce the accuracy of diagnosis [283], which places greater demands on the comprehensiveness and versatility of the nano-theranostics. More specifically, whether the efficacies of metal ions are affected by the characteristics of TME also needs to be considered [284]. For instance, many metal ions induce tumor cell death by generating reactive oxygen species. However, it seems that the anoxic and highly reductive state of TME is disadvantageous to the process. How to ensure appropriate pH and adequate concentration of H_2_O_2_ to activate the catalytic pathway is worth pondering. Further studies are needed to determine whether specific immunoregulatory mechanisms in different tumor types resist the strategies that activate immune pathways by metal ions. Besides, there is an urgent need for research on mechanisms in point of the new approaches, such as ferroptosis, calcicoptosis, and especially cuproptosis, which makes it possible to achieve broader anti-cancer applications.

Ultimately, controlled clinical trials are mandatory to define better the limits and effectiveness of this promising novel diagnostic and therapeutic tool. Over time, metal ions are expected to become more powerful weapons against cancer.

## Data Availability

Not applicable.
